# Supplementary material for: Deep learning-based classification of peptide analytes from single-channel nanopore translocation events
Source: PLoS One. 2025 Sep 11;20(9):e0324777. doi: 10.1371/journal.pone.0324777 (PMC12425215; doi:10.1371/journal.pone.0324777)
Supplement: Attachment — Submitted filename: Response to Reviewers.pdf [file pone.0324777.s001.pdf]

Editor's Comments/Journal Requirements:

1. **[Editor/Journal Requirement 1]** Ensure the manuscript meets PLOS ONE's style requirements, including file naming. (Refer to provided templates).

   I added Headings Styles in all the proper places and followed other stylistic conventions to the manuscript by the Style Guidelines.

2. **[Editor/Journal Requirement 2]** Ensure all author-generated code is made available without restrictions upon publication, following PLOS ONE's code sharing guidelines. Provide the GitHub link for reviewers/readers.

   The author-generated code was added to GiHub at the url (https://github.com/bakrantz/Pept-Class). Also note that the experimental and simulated datasets (labeled stream .csv and processed events .pkl files) are contained there alongside the Python code.

3. **[Editor/Journal Requirement 3]** Amend the financial disclosure statement to explicitly state the funders' role in the study (e.g., "The funders had no role in study design, data collection and analysis, decision to publish, or preparation of the manuscript." or an accurate alternative). Include this amended statement in the cover letter.

   I included the funding statement in the cover letter.

4. **[Editor/Journal Requirement 4]** Remove all funding-related text from the Acknowledgments section or other areas of the manuscript. Confirm the preferred updated Funding Statement in the cover letter.

   I removed funding-related text from the Acknowledgments.

5. **[Editor/Journal Requirement 5]** Complete the Competing Interests on the online submission form. If no competing interests, state "The authors have declared that no competing interests exist." in the cover letter.

   I added the statement: “The authors have declared that no competing interests exist” to the cover letter.

6. **[Editor/Journal Requirement 6]** Clarify whether the adapted/rescaled/reoriented figures (Fig. 1A and 1B) were from previously peer-reviewed and formally published work. If so, provide the reason in the cover letter why this does not constitute dual publication.

   Fig 1 is a background/introduction figure, which is purely pedagogical, and does not constitute the main examined Results data and findings of this manuscript. Instead of re-using the bitmap in that figure, I re-generated a new bitmap in Fig 1A with slightly different orientation and colors. Therefore, the purpose of the image is intact but there are no re-use/licensing concerns for PLOS. In Fig 1B, instead of re-plotting earlier published data, I decided to re-make the figure with new peptide translocation events samples, thus preserving the pedagogical purpose of showing that a nanopore system

indeed populates several intermediate conductance states. Hopefully this satisfies any lingering concerns.

7. **[Editor/Journal Requirement 7]** Decide on a data sharing plan now, rather than waiting for acceptance. Ensure that data will be made freely accessible upon publication, explaining any necessary access restrictions or seeking an exemption if public deposition is not feasible.

   As I stated above regarding the generated code deposition at GitHub, all datasets (both simulated and experimental) are deposited at GitHub (https://github.com/bakrantz/Pept-Class). This will be more convenient to have data and code together.

8. **[Editor/Editor Comment]** Add a brief discussion on the broader biomedical relevance of peptide analyte classification, including its roles as therapeutics, biomarkers, and contributors to various diseases (metabolic, neurodegenerative, infectious, immune modulation).

   I created a new subsection at the start of the Discussion to frame the broader impacts.

Biomedical impact of peptide classification

Beyond fundamental research into peptide characteristics, the accurate and rapid classification of peptides holds significant biomedical relevance. Peptides are increasingly recognized for their therapeutic potential [1]. They serve as crucial biomarkers in a wide array of diseases, including various forms of cancer [2]. Moreover, specific peptides are emerging as potential therapeutics, for instance, those used to treat neurodegenerative diseases [3], or as critical anti-microbials that combat infectious diseases, such as tuberculosis [4]. Furthermore, peptides play pivotal roles in immune modulation, acting as signaling molecules or therapeutic agents that can influence immune responses [5-7]. The existence of vast databases compiling key peptide biomarkers and therapeutic peptides across these fields [1, 2, 4, 5] underscores their invaluable resource in the development of new detection and therapeutic methods. The ability to precisely identify and differentiate individual peptides, as demonstrated by our nanopore-based classification method, is therefore a foundational step towards early disease detection, targeted therapeutic development, and personalized medicine. Our approach presents a promising platform for high-throughput peptide analysis that could contribute to advancing these critical biomedical applications.

Note the citations are of course different numbers in the text of the manuscript. They are enumerated here for your reference:

1. Usmani SS, Bedi G, Samuel JS, Singh S, Kalra S, Kumar P, et al. THPdb: Database of FDA-approved peptide and protein therapeutics. PLoS One. 2017;12(7):e0181748. Epub 20170731. doi: 10.1371/journal.pone.0181748. PubMed PMID: 28759605; PubMed Central PMCID: PMCPMC5536290.

2. Bhalla S, Verma R, Kaur H, Kumar R, Usmani SS, Sharma S, et al. CancerPDF: A repository of cancer-associated peptidome found in human biofluids. Sci Rep. 2017;7(1):1511. Epub 20170504. doi: 10.1038/s41598-017-01633-3. PubMed PMID: 28473704; PubMed Central PMCID: PMCPMC5431423.
3. Usmani SS, Jung HG, Zhang Q, Kim MW, Choi Y, Caglayan AB, et al. Targeting the hypothalamus for modeling age-related DNA methylation and developing OXT-GnRH combinational therapy against Alzheimer's disease-like pathologies in male mouse model. Nat Commun. 2024;15(1):9419. Epub 20241031. doi: 10.1038/s41467-024-53507-8. PubMed PMID: 39482312; PubMed Central PMCID: PMCPMC11528003.
4. Usmani SS, Kumar R, Kumar V, Singh S, Raghava GPS. AntiTbPdb: a knowledgebase of anti-tubercular peptides. Database (Oxford). 2018;2018. doi: 10.1093/database/bay025. PubMed PMID: 29688365; PubMed Central PMCID: PMCPMC5829563.
5. Usmani SS, Agrawal P, Sehgal M, Patel PK, Raghava GPS. ImmunoSPdb: an archive of immunosuppressive peptides. Database (Oxford). 2019;2019. Epub 20190101. doi: 10.1093/database/baz012. PubMed PMID: 30753476; PubMed Central PMCID: PMCPMC6367516.
6. Nagpal G, Usmani SS, Dhanda SK, Kaur H, Singh S, Sharma M, et al. Computer-aided designing of immunosuppressive peptides based on IL-10 inducing potential. Sci Rep. 2017;7:42851. Epub 20170217. doi: 10.1038/srep42851. PubMed PMID: 28211521; PubMed Central PMCID: PMCPMC5314457.
7. Dhall A, Patiyal S, Sharma N, Usmani SS, Raghava GPS. Computer-aided prediction and design of IL-6 inducing peptides: IL-6 plays a crucial role in COVID-19. Brief Bioinform. 2021;22(2):936–45. doi: 10.1093/bib/bbaa259. PubMed PMID: 33034338; PubMed Central PMCID: PMCPMC7665369.

Reviewer #1 Comments:

1. **[Reviewer #1, Item 1]** Discuss or elaborate on how large datasets are crucial for deep learning models and how they enhance effectiveness during training and validation.

   Thank you for highlighting this fundamental principle, which we alluded to in the introduction. I fully agree, and I decided to at first use simulated data to generate a large and controlled dataset rather than use a smaller existing sample size of experimental data; increased data volume is critical for training robust deep learning models. We added some phrasing to the manuscript in the Methods and Discussion to further emphasize this concern in our study design.

   In the Methods: “Simulated data was chosen in this study to have a large enough controlled dataset to train robust deep learning models and facilitate their development.”

   In the Discussion: “Here we used a large enough controlled, simulated dataset to aid in proper model development and training, thus increasing the chances of success on adapting this approach to real-world nanopore translocation data.”

2. **[Reviewer #1, Item 2]** Discuss or elaborate on how increasing the number of layers in a neural network can improve predictive accuracy by learning more intricate patterns.

The model architecture (TCN-Dense) settled on was designed with a specific number of layers chosen after preliminary architectural exploration to balance complexity, training stability, and performance. The training time was also optimized by reducing dilations and filters without sacrificing evaluation metrics and performance. There are trade-offs in increasing model complexity (e.g., longer training times and overfitting risks).

We added this sentence to the methods explaining how we settled on the number of TCN blocks and layers as well as other key parameters: “The number of TCN blocks, dilations, filters and kernel size were chosen to balance the ability to maintain evaluation metrics while reducing computational costs.”

3. **[Reviewer #1, Item 3]** Ensure proper implementation and discussion of dropout regularization in the Keras model.

   The dropout_rate parameter was applied in the TCN blocks at a level of 0.3. Dropout regularization was also applied in the Dense layers at a level of 0.3. These dropouts were specified in the Methods section. We added a statement to the Methods reflecting that dropout regularization reduces overfitting.

4. **[Reviewer #1, Item 4]** Discuss or elaborate on the role of increasing epochs in training and how it can improve accuracy.

   We trained the models for a sufficient number of epochs, employing callbacks of early stopping with a patience to prevent overfitting and ensure optimal learning without unnecessary computation. The maximum number of epochs allowed was 30 for TCN-Dense model. The validation metrics were largely plateaued at that point. No further optimization of epoch number was performed.

   The manuscript describes our implementation of this in the Methods: “Training was conducted with a batch size of 32 for 30 epochs, with 20% of the training data reserved for validation. To prevent overfitting and optimize training, early stopping (patience of 20 epochs), model checkpointing (saving the best weights based on validation loss), and a learning rate reduction on plateau (factor of 0.5, patience of 5 epochs, minimum learning rate of 10^{-5}) were implemented as callbacks during the training process.”

5. **[Reviewer #1, Item 5]** Discuss hyperparameter optimization as an iterative process, mentioning systematic experimentation with learning rates, batch sizes, and epochs, and recommending tools like grid search or random search for efficient exploration.

   We acknowledge the reviewer's emphasis on systematic hyperparameter optimization. While we did not employ an automated grid search or extensive random search for all hyperparameters, our model development involved an iterative and principled manual tuning process. We systematically explored the impact of key architectural choices, including TCN block arrangements, filter numbers, dilation rates, and kernel sizes, on model performance and stability. Similarly, we fine-tuned the dropout rate by observing its effect on overfitting and learning efficiency. We basically moved dropout until it went from the extreme of overfitting to the other extreme of not learning effectively, striving for good balance. This iterative approach allowed us to identify the optimal parameter

combinations that yielded the robust performance reported in our study. We agree that formal search methodologies could be beneficial for even broader parameter space exploration in future investigations.

We added the following clarification of our more manual parameter search process to the Methods section: “The selection of the TCN/Dense architecture's specific parameters, including the number and configuration of TCN blocks, filter counts, dilation rates, and kernel sizes, as well as the dropout rate, was refined through an iterative manual tuning process. This involved systematic experimentation and evaluation on the validation dataset to optimize for classification performance and training stability.”

Reviewer #2 Comments:

Major Comments:

1. **[Reviewer #2, Major 1]** Investigate and discuss alternative strategies or model adjustments to enhance event-level classification accuracy in peptide mixtures (e.g., better feature engineering, alternative network architectures, or confidence-threshold optimization).

   We appreciate the reviewer's insightful comment regarding the moderate event-level classification accuracy (0.70) for peptide mixtures. We acknowledge that this is a key area for improvement and have thoroughly investigated potential strategies and model adjustments. We've added a dedicated section in the Discussion to detail these avenues, outlining their mechanisms and our preliminary thoughts or prior findings, and framing deeper explorations as important future work, particularly with real nanopore datasets.

   Here is a breakdown of the areas suggested by the reviewer to explore as well as another area we have been investigating more recently:

   A. Enhanced Feature Engineering. We agree that expanding our feature set holds significant promise. As discussed in the manuscript, the inclusion of scaled current levels of observed conductance states, which are known to vary between guest-host peptides in real PA nanopore data, could provide richer information. Our simulated three-state data, while demonstrating feasibility, represents a simplified scenario. Generalizing to peptides that populate four or more conductance states in real systems would naturally introduce more discriminative features that could enhance mixture classification.

   B. Alternative Network Architectures. While our current manuscript details the stability advantages of TCN-Dense over LSTM-Dense, we recognize that other architectures could be beneficial. We have considered and, in some preliminary testing since initial submission, explored CNN-Dense architectures for scaled current traces of the translocation events. While powerful and often faster to train, they can be more susceptible to noise. A particularly promising future direction is the application of 1D CNNs directly to the state sequences themselves. This would allow the model to learn local patterns within the state transitions, which could be highly informative.

C. Confidence-Threshold Optimization. The confidence threshold is a user-defined parameter in our event-level mixture prediction script. We did perform some initial exploration of this parameter and found that while it modestly affected predictions, extremely selective thresholds did not universally lead to more faithful predictors across all mixtures. This suggests that the primary limitation may lie in the inherent information content of individual short events or the features themselves, rather than merely the threshold. A more in-depth, systematic optimization would be warranted in future studies.

D. Event-Length Filtering. Although not explicitly detailed in the initial submission, our subsequent preliminary investigations have shown that filtering out very short-duration events can indeed improve classification accuracy and F1-score across various neural network and traditional machine learning models. Conceptually, short events contain less information. While effective, this approach must be applied judiciously, especially for peptides with fast kinetics where many informative events could be inadvertently removed. We deem this a critical strategy to explore in greater depth as part of our follow-up study on real nanopore event datasets, where event duration distributions are empirically known.

These strategies collectively offer compelling avenues for enhancing event-level classification accuracy in complex peptide mixtures, forming a significant part of our future research.

Because this is an important topic. We included a section in the Discussion, delineating these research prospects in improving event-level predictions of mixed samples.

“**Exploiting methods to enhance peptide mixture classification**

While the deep learning framework presented here demonstrates high accuracy for classifying pure peptide streams and shows promising capability for peptide mixtures, the event-level classification accuracy (~0.70) indicates room for further improvement. Enhancing performance in this challenging area is critical for practical biosensor applications. We envision four strategies and model adjustments to address this: (i) feature engineering, (ii) exploring alternative neural network architectures, (iii) confidence-threshold optimization, and (iv) event-length filtering.

For feature engineering, future work will investigate the inclusion of additional, potentially highly discriminative, features. For instance, the exact scaled current levels of the observed conductance states, which are known to vary uniquely among different guest-host peptides in real PA nanopore data, could provide additional richer information. Furthermore, while our simulated data was simplified having only three conductance states, real guest-host peptides are known to populate four or more distinct states. Generalizing our approach to incorporate these higher numbers of states would naturally expand the feature space, potentially leading to more robust classification.

With regard to exploring alternate network architectures, initial investigation focused on TCN/Dense, chosen for its stability compared to LSTM/Dense. However, other architectures warrant exploration. CNNs, for example, may be applied directly to the scaled current traces of events and may offer powerful pattern recognition capabilities. While potentially faster to train, 1D CNNs can be more susceptible to the

inherent noise in raw nanopore current data. A particularly promising avenue is the application of 1D CNNs directly to the pre-classified conductance state sequences. This approach would allow the model to learn local and contextual patterns within the sequence of discrete states, which may encode unique peptide-specific signatures that are not fully captured by aggregate features. These CNN models can also incorporate features in a concatenated Dense input layer analogous to the TCN-Dense model featured in this study.

The current event-level mixture prediction utilizes a user-defined confidence threshold. While some preliminary testing was conducted, an exhaustive, systematic optimization of this parameter across various mixture complexities and noise levels could refine the overall mixture classification accuracy. However, initial observations suggest that the primary limitation may stem from the inherent information content within very short or ambiguous individual events, rather than merely the threshold setting itself.

The current analysis did not explicitly exploit event-length filtering as a primary optimization strategy. However, we have observed in preliminary investigations that discarding translocation events below a certain duration can significantly improve classification accuracy and F1-score across various machine learning and deep learning models. This is conceptually aligned with the idea that extremely short events contain less information content, making them difficult to classify reliably (analogous to classifying an image with very few pixels). While beneficial for enhancing accuracy, this strategy must be applied judiciously, particularly for peptides known to exhibit rapid kinetics, as it could inadvertently remove a substantial portion of potentially classifiable events. A detailed study on the optimal application and trade-offs of event-length filtering will be a crucial component of our follow-up research utilizing real PA nanopore data."

2. **[Reviewer #2, Major 2]** Provide additional details on the feature selection strategy, including how global and event-level features were determined. Elaborate on the instability with the variance feature, potentially with supplementary analysis (e.g., ablation studies).

   We could have been better about describing the features set we used in more detail during our feature engineering phase. I now have included a detailed list of them in the Methods, divided into the two categories: event-level features and global features. They are, of course, delineated in the source code that specifically segments the raw state labeled translocation streams (.csv file) into individual translocation events, During segmentation the script computes features for the events at the event-level and globally for the entire stream's events (output as a .pkl file). These translocation events (state sequences and the features) are saved as pickle files as a list of dictionaries, and they become the training data used in the models.

   Here is the text I added to the Methods delineating the entire feature set:

   "The initial set of event-level features included: state sequence entropy, first transition time, average dwell in state 0, average dwell in state 1, variance of dwell in state 0, variance of dwell in state 1, longest dwell in state 0, longest dwell in state 1, event duration, probability in state 0, probability in state 1, ratio of probabilities in state 0 to state 1, and number of transitions. Note we removed entropy from this initial event-level features set when permutation importance analysis showed that its removal did not

reduce F1-scores. The initial set of global features included: average of event duration, variance of event duration, average event entropy, average first transition time, average number of transitions, overall probability in state 0, overall probability in state 1, and overall ratio of probability in state 0 to state 1. The global feature, variance of event duration, was removed when it significantly degraded performance, as described in the Results."

We did two things regarding the feature selection strategies. First, we noticed serious performance losses when variance of event duration was included as a global feature. We do not know why this occurred, but we removed each global feature one at a time to isolate the feature(s) that degraded performance. As we say in the text, "a significant degradation in model performance, evidenced by a drop in overall accuracy from 0.99 to 0.60 and a decrease in macro-averaged F1-score from 0.99 to 0.55, indicating that this feature introduced noise and model instability into the classification." The second thing we did was permutation importance analysis, and we found that state sequence entropy did not really contribute to improving F1-score, so we removed it as an event-level feature. We noted this removal in the Methods section above. It probably would not matter much if it was included, since it did not affect F1-score upon removal. This is unlike the global feature, variance of event duration, which was very pathological.

3. **[Reviewer #2, Major 3]** Discuss alternative methods for class balancing, such as data augmentation or synthetic data generation, given the importance of balanced classes and the brief mention of downsampling.

   I added the following text, summarizing our approach and future directions on class balancing: "For the present work, downsampling was selected to provide a clear and controlled demonstration of our model's capabilities with balanced, experimentally-relevant event counts, prioritizing the fidelity of the simulated data. In future work with real datasets, we may explore data augmentation of the raw current traces and apply SMOTE or ADASYN. This may potentially leverage all available data and further enhance the robustness and generalization capabilities of our classification framework, especially when dealing with naturally imbalanced real-world nanopore datasets."

4. **[Reviewer #2, Major 4]** Justify the choice of TCN over LSTM more fully with quantitative comparison data or figures illustrating performance/stability differences.

   We initially tried feeding the state sequence into an LSTM and concatenating that output with the set of features which were fed into a Dense branch, but there was reproducible instability in the validation accuracy and macro-averaged F1-score metrics. When we switched to TCN for the state sequence input, the instability resolved. In retrospect, knowing what we know now, we should have also thoroughly tested a multi-layer 1D CNN as well. We have since done that and there is some performance boost, but it is probably beyond the scope of this study to include all possible NN's for the states sequence input.

   Below includes figures of the training for a LSTM implementation we used. To account for this issue with the LSTM we made a note in the manuscript that validation test set accuracy and macro-averaged F1-score were unstable with that architecture.

The text includes the description of LSTM instability and better performance with the TCN: “When an LSTM network was employed in place of the TCN, the model demonstrated severe validation volatility, with divergent validation behavior, as shown by both accuracy and macro-averaged F1-score, compared to the training data. This instability prevented convergence and led to poor generalization, ultimately motivating the adoption of the TCN architecture.”

Figure of LSTM-Dense model showed instability in validation accuracy and F1-score.

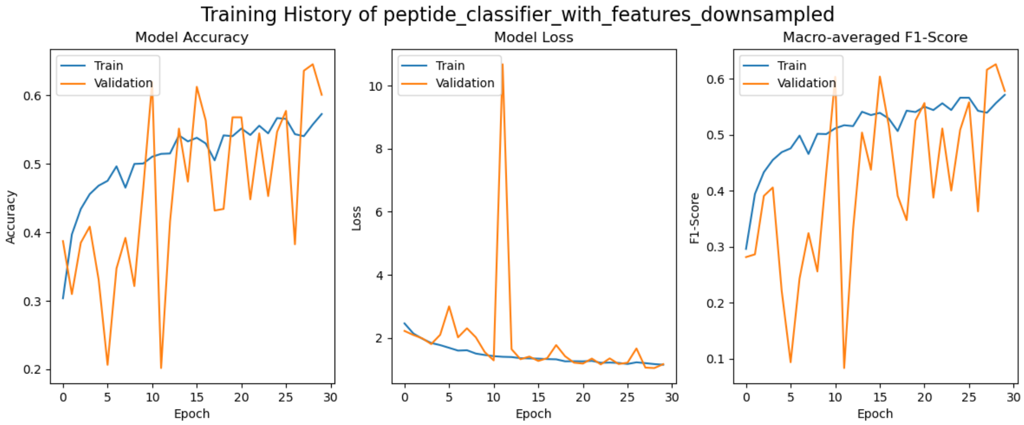

Example figure of TCN-Dense model showed much less instability when trained on state sequences and event-level features.

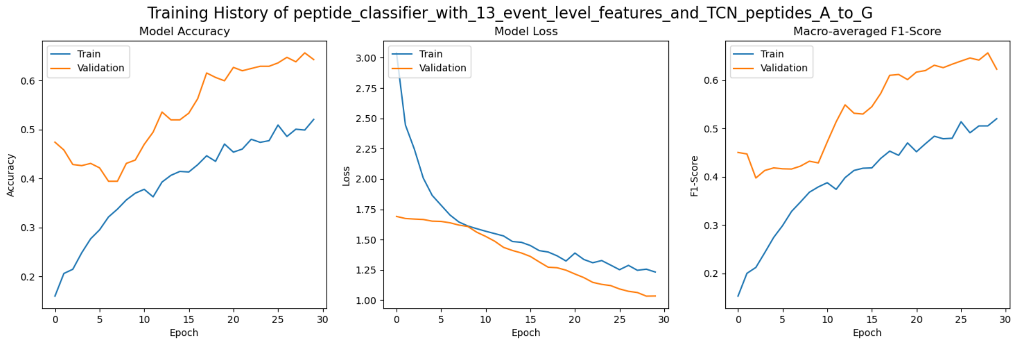

The create model function in the training script can select whether the user wants to use the TCN, LSTM, or even both, so it could be easily explored in follow-up analysis. We just decided to not pursue the matter much further, since the issue resolved itself with use of a TCN. It is entirely possible an alternate configuration of an LSTM would be more stable than our initial implementation. We just “back burnered” that exploration for the time being.

Minor Comments:

1. **[Reviewer #2, Minor 1]** Clarify the legend for Fig. 2 (Page 33) by explicitly labeling color codes or state numbers.

Color names were annotated in the Figure 2 legend text. The enumerated states were given biophysical meaning in the figure legend: i.e., fully peptide-blocked nanopore is state 0; partially blocked nanopore is state 1; and fully open nanopore is state 2.

2. **[Reviewer #2, Minor 2]** Consider providing numerical accuracy or F1-scores within the confusion matrices in Fig. 4 (Page 35) for greater clarity.

   I updated the Fig. 4 legend by inserting the accuracy and F1-score values (mean and std. deviations) corresponding to the two different confusion matrices.

   "**Fig 4. Confusion matrices for TCN/Dense model peptide classifications from translocation events.** Peptides A-G are indicated when using the following inputs **(A)** conductance state sequences with global- and event-level features as input (overall accuracy of 0.9998 (±0.0006) and macro-averaged F1-score of 0.9998 (±0.0006)) or **(B)** conductance state sequences with only event-level features as input (overall accuracy of 0.70 (±0.01) and macro-averaged F1-score of 0.70 (±0.02)). The best matrix out of six replicates for either type of training is shown."

3. **[Reviewer #2, Minor 3]** Ensure the GitHub link for source code, simulated datasets, and real datasets is clearly provided on page 7.

   The generated code is deposited at GitHub (https://github.com/bakrantz/Pept-Class) and both the simulated and experimental datasets (.csv files and .pkl files) used in that code are in the same location for ease of downloading and re-analysis. We included this link in the text now on page 7, since the repository is functional now and set up.

4. **[Reviewer #2, Minor 4]** Consistently include confidence intervals or statistical significance for reported accuracy, precision, recall, and F1-score to improve scientific rigor.

   We thank the reviewer for this important suggestion regarding statistical rigor. We agree that including measures of variability enhances the robustness of our reported metrics.

   To address this, we have now performed six independent training runs for the TCN-Dense model for both the pure peptide classification and the peptide mixture classification tasks. For each replicate, different random_state seeds were used for the data splits to assess consistency and variability across distinct data partitions.

   a. Pure Peptide Classification (Global and Event-level Features):

   For the task of classifying pure peptide streams, where our model consistently achieves exceptionally high performance, the results of the 6 replicates are as follows (Mean ± Standard Deviation):

   Overall Accuracy: 0.9998±0.0006
   Macro-averaged Precision: 0.9998±0.0005
   Macro-averaged Recall: 0.9998±0.0006
   Macro-averaged F1-score: 0.9998±0.0006

These quantitative results demonstrate that the model's performance for pure peptide classification is remarkably stable and near-perfect across different runs. We attribute this high performance to the distinct and clean nature of the simulated single-peptide translocation events and the robust feature representation provided by the combined global and event-level features. We have updated the locations in the manuscript to reflect these mean and standard deviation values. A representative confusion matrix, showing perfect classification for a typical run, has also been included/updated in Figure 4.

b. Peptide Mixture Classification (Event-level Features Only):

For the more challenging task of classifying peptide mixtures using only event-level features, the results of the 6 replicates are as follows (Mean ± Standard Deviation):

Overall Accuracy: 0.7008±0.0134
Macro-averaged Precision: 0.7098±0.0208
Macro-averaged Recall: 0.7004±0.0134
Macro-averaged F1-score: 0.6972±0.0160

These quantitative results demonstrate that while there is some natural variability, the model's performance for peptide mixture classification remains consistently within a narrow range across different data splits, underscoring its robustness. We have updated the relevant table in the manuscript to reflect these mean and standard deviation values.

For future work, particularly with larger, real-world datasets, we are committed to conducting a broader statistical analysis, including confidence intervals derived from multiple training runs, to further quantify model variability across all configurations.

5. **[Reviewer #2, Minor 5]** Check the consistency of formatting (e.g., space between "Deep-Channel" and subsequent terms).

   I believe “Deep-Channel” with the hyphen is the GitHub project name and the name used in their manuscript. I found 11 instances of that exact formatting in our current manuscript. I just mirrored that hyphenated name format in my reference to their work. The exact title of their paper is as follows: “Deep-Channel uses deep neural networks to detect single-molecule events from patch-clamp data.” There are no other versions like “DeepChannel” (no space) or “Deep Channel” (space) in our manuscript (using Find in MS Word).

6. **[Reviewer #2, Minor 6]** Briefly discuss the scalability or potential limitations of the pipeline in practical scenarios involving significantly larger datasets, given the highlighted performance gain from hardware upgrade.

   We’ve collected much larger real datasets of single-channel translocation event streams for anthrax toxin PA nanopore using guest-host peptides, and we have run into issues with handling large numbers of files of various conditions and their replicates for seven different peptides, so we created a pair of databases to be able to access the datasets with the proper set of experimental parameters and event segmentation and feature

calculating processing parameters. In exploring real data, we’ve found that the TCN-Dense model does train slower than other CNN-Dense models we recently have employed. So, there are real-world issues with processing speed when comparing neural network architectures. Like I said earlier, knowing what we know now, we would have explored CNN-Dense more extensively in the simulated set of Peptides A-G due to the processing speed of that model. Even faster is the machine learning tree-based classifier, XGBoost, using only the feature set. We’ve decided to report on these practical issues involving larger real datasets in the follow up paper we are working on now.

7. **[Reviewer #2, Minor 7]** Carefully read the manuscript and rectify grammatical and punctuation issues.

   We proofread the revised draft now. Hopefully we found everything.
